# A Short History of Vitriolic Æther, with an Account of Its Peculiar Efficacy in Gouty Affections of the Stomach; and an Attempt to Investigate Hoffman's Genuine Method of Preparing His Anodyne Mineral Liquor

**Published:** 1785

**Authors:** 


### LONDON MEDICAL JOURNAL,

FOR THE YEAR 1785,

### PART THE FOURTH.

### SECTION I.

### ESSAYS AND OBSERVATIONS.

I. *A ſhort Hiſtory of Vitriolic Æther, with an Account of its peculiar Efficacy in gouty Affections of the Stomach; and an Attempt to inveſtigate Hoffman's genuine method of preparing his Anodyne Mineral Liquor. By Mr.* William Tickell, *Apothecary, at Bath. Communicated in a Letter to Sir* Joſeph Banks, *Bart. P. R. S. and by him to Dr.* Simmons.

ÆTHER is the lighteſt, moſt volatile, and inflammable of all known fluids; it ſwims on the moſt highly rectified ſpirit of wine; and, dropped on the warm hand, almoſt inſtantly exhales, diffuſing a penetrating fragrance, and leaving no moiſture behind. — It does not mingle with water, with acid or

 alkaline alkaline liquors, but diſſolves ſome unctuous and reſinous bodies. It has a greater affinity with gold than the other eſſential oils; for if a ſolution of gold in aqua regia be mixed with æther, it will keep the gold ſuſpended.— Its inflammability may be attributed to the volatility and extreme tenuity of its oily parts.

Valerius Cordus is the firſt author who deſcribes the proceſs for making æther. This he does in a very perfect manner, and indeed his deſcription is ſo explicit and particular, that perhaps a more clear and circumſtantial account can ſcarcely be found in the works of any ſubſequent writer. The ſame proceſs was afterwards copied by Conradus Geſnerus, and publiſhed about the year 1552. From that time the preparation ſeems to have been neglected, or rather totally unknown for more than a century; and, amongſt the moderns, Frobenius, a German, is conſidered as the firſt who brought it to perfection; and ſome have even attributed the invention of it to him. In this number I may reckon M. Macquer, who denies that the ancient chymiſts were acquainted with it; or, if they were, remarks, that their deſcription was couched in ſuch enigmatical terms as rendered the ſubject altogether unintelligible:

telligible: a cuftom indeed which pretty generally prevailed with the alchymifts. He likewife obferves, that Meffrs. Duhamel, Groffe, and Hellot, who were at great pains in making a variety of experiments on this fubject, and have communicated to the public an exact and certain method of making æther, having received no fuccours in their labours but what were derived from their own penetration and fagacity, have alfo a right to claim the merit of the invention. At the fame time he acknowledges, that other chymifts knew how to make æther, particularly Boyle and Newton; but thefe great philofophers never gave any particular procefs. Perhaps the author above mentioned never fell into the hands of thefe gentlemen; as the celebrated Fred. Hoffman fays that he hit on a fimilar procefs before he had feen that defcribed by Valerius Cordus.

M. Macquer has given the moft plaufible theory of the production of this fubtil fluid, which he fuppofes to be nothing more than fpirit of wine partly decompofed by the vitriolic acid; that is to fay, in a great meafure deprived of its phlegm or watery part, and its oily part extremely fubtilized: hence it becomes immiſcible with water, and, in ſome ſort, approaches the nature of an eſſential oil.

It is probable, that if the effects of æther were properly attended to, it would appear to be of more importance in medicine than is generally ſuppoſed; though it muſt be owned that its action is not always equally certain; when it ſucceeds, its effects are ſudden — it operates like a charm. In violent hyſteric fits, a few drops, taken internally, will often give ſudden relief; and in pains of the head, ariſing from ſpaſm, a little æther poured into the hollow of the hand, and applied to the part, has frequently produced a cure. I have alſo ſeen the tooth-ach immediately relieved by it; and in fixed rheumatiſms, when externally applied, much benefit may be expected from it. But I have known it do very eſſential ſervice in a complaint which is of the moſt alarming and dangerous kind, and, in ſome inſtances, where every other medicine which had been tried failed of ſucceſs — I mean the gout in the ſtomach *. I ſhall endeavour to confirm this aſſertion

* Æther will be more particularly ſerviceable, when, either from debility of the patient, ſome unforeſeen accident or miſmanagement, a metaſtaſis of the gouty humour has taken place;

ſertion by giving the hiſtory of a very remarkable caſe; and am alſo happy to find that the ingenious Dr. Lind has lately adminiſtered it with great ſucceſs to ſeveral of his patients labouring under a ſimilar complaint*.

CASE I.

The Rev. T. H. rector of a pariſh in the weſtern part of Devon, had naturally a good conſtitution and firm fibre; but, unhappily, at the age of eighteen years, he was attacked by a ſevere fit of the gout, which, notwithſtanding the opinion of a learned author, I ſhall call hereditary, as his mother and ſeveral of his family in the female line were early martyrs to it. His phyſician, at the concluſion of the paroxyſm, adviſed him to begin a courſe of muſk, which was continued for a conſiderable time with the viſionary hope of totally eradicating the gouty acrimony: however, at the end of about twelve months, he was viſited by another place; for when the gout in the ſtomach is occaſioned by a redundancy of gaſtric juice, which, from a long ſtay, is become vitiated, or from a ſluggiſh, inert, or acrid bile, an emetic may be adminiſtered with the greateſt ſafety and ſucceſs, and æther will afterwards more powerfully exert its ſedative and antiſpaſmodic virtues.

* See page 53.

ther attack of the gout; and this periodical return continued for fome years during the vernal months. The fits afterwards were more frequent, more fevere, irregular, and of longer duration. The gouty humour had now made a depofit on different joints of the fingers and toes; and, from feveral of the fores, there was a continual difcharge of chalky matter. The tone of the ftomach and bowels appeared alfo to be confiderably affected; and a ficknefs, naufea, and urging to vomit, now generally preceded every gouty paroxyfm. His bowels were, moreover, almoft conftantly in a lax ftate. During the intervals, he had great fpirits and tolerable health, which was affifted probably by a conftant ufe of exercife on horfeback; but being fond of hilarity and good company, he did not always pay that due regard to regimen, and attention to the non-naturals, which is fo neceffary to be obferved in gouty conftitutions, and which, perhaps, would have been the only means of his acquiring any degree of permanent benefit.

During the fummer months of the year 1761, and a great part of the fubfequent winter, he was confined to his bed, or, at leaft, to the houfe, by feveral fucceffive fits of the gout; and and there was ſo great a redundancy of gouty acrimony, that notwithſtanding a conſiderable degree of external gout, attended with a good deal of inflammation, and the diſcharge already mentioned, yet both the head and ſtomach were at times very much affected. He was, therefore, the following ſpring adviſed to make trial of the Bath waters, with a view of ſtrengthening the tone of his ſtomach and bowels, bracing the general habit, and aſſiſting nature in her future efforts to expel the gouty humour. He viſited Bath accordingly, and having drank the waters ſparingly for the ſpace of three weeks, was ſenſible of the approach of a gouty paroxyſm, which proved to be the moſt ſevere external and univerſal gout he had ever experienced, as it affected almoſt every member of the body, not excepting the ears and noſe. Though a few days after the atrack he had a very good appetite, he could ſubmit to be fed only with a tea ſpoon, and the thinneſt diet, as panada, ſago, broths, &c.; nor did he attempt to take ſolid food, the action of chewing being abſolutely prevented.

The fit laſted ſome weeks; but on his return to the country air, with a conſtant uſe of horſe exerciſe, he was ſo much recovered, and his conſtitution conſtitution ſo much invigorated, that for nearly two years afterwards his fits of the gout were ſo regular and moderate as to require little or no medical aſſiſtance. At the expiration of that time, however, the complaints of the head and ſtomach began to return, and plainly indicated the propriety of another journey to Bath; but either from buſineſs, or a reluctance at leaving home, he could not be prevailed on to give the waters another trial.—In the month of April, 1764, he became low ſpirited, with loſs of appetite; complained of head-ach in the morning, with frequent tendency to vomit; had reſtleſs nights, and was conſtantly very ill till after dinner; when, by the aſſiſtance of a few glaſſes of generous wine, his ſpirits were for ſome hours relieved.

Early in the morning, on the 5th of May, having paſſed a very troubleſome and reſtleſs night, he was ſeized with a violent and excruciating pain in the ſtomach, accompanied with ſickneſs; the efforts in vomiting were moſt ſevere and painful, and his head was violently affected. Theſe ſymptoms were attended with a conſiderable degree of thirſt, a lax ſtate of the bowels, and pale crude urine: his pulſe was low, quick, and depreſſed; and he complained plained of ſo great a degree of anxiety and oppreſſion at the præcordia as ſeldom happens but in the moſt dangerous diſeaſe, and in the moſt alarming caſes.

An emetic was adminiſtered, which gave only temporary relief; then the warmeſt cordials, joined with opiates, with as little ſucceſs, as they were very ſoon rejected. Uſquebaugh and burnt brandy, in which tanſey and mint had been infuſed, were alſo had recourſe to. Warm ſtimulating plaſters were applied all around the feet; and after the ſtomach had been ſeveral times fomented with brandy, a veſicatory was applied over that region. Neither medicine, food, nor any kind of cordial remained on the ſtomach more than a few minutes; and every effort to vomit occaſioned an extreme increaſe of pain. He had now had no ſleep for nearly forty-eight hours, and ſcarce any reſpite from pain during that time. In ſhort, I was never more diſtreſſed on account of the very violent and unalleviated ſufferings of any patient. I had tried every medicine that had procured eaſe on former emergences, without the leaſt ſucceſs; and the extreme violence of the diſeaſe made me deſpair of giving any relief.— The great diſtance from a phyſician made it

 dangerous dangerous to wait, and humanity required ſome-thing to be immediately done. In this di-lemma it occurred to me, that vitriolic æther, from its ſedative power, and by exerting its action principally on the ſtomach, might poſſi-bly afford ſome mitigation of the more-alarm-ing ſymptoms, though I had not then ever known it given or even recommended in a ſimi-lar caſe. I began with twenty drops in a little peppermint water. This remained on the ſto-mach, which was gaining a conſiderable point, and it encouraged me to repeat the doſe, which I did in the ſpace of half an hour, increaſing it to thirty drops, which procured the patient ſome eaſe, and of courſe he became leſs reſt-leſs. In another hour I gave forty drops, which procured not only eaſe but alſo half an hour's ſleep. In ſhort, I gradually increaſed the doſe to ſixty drops, giving it at longer in-tervals, till he became perfectly eaſy and had obtained ſome hours ſleep; and at noon* he was ſo far recovered as to be deſirous of taking ſome food. Forty drops of æther, with a few grains of cardamom ſeed in powder, and ſome peppermint water, compoſed a draught which

* May 6.

was was given every ſix hours for five or ſix days, when the patient appeared to be perfectly recovered.

It is not a little ſingular in this gentleman's caſe, that he received a perfect cure by the above method, without any degree of gout forming in the extremities; from which circumſtance we are diſpoſed to infer, that this very volatile and penetrating fluid has not only the power of diſlodging the ſubtile gouty humour from the more noble viſcera, but likewiſe of eliminating, and, as it were, by a kind of electric property, diſpelling it from the habit through the cutaneous pores.

A ſpecific in this diſeaſe is a deſideratum in medicine, as the moſt powerfully warm and cordial remedies, aſſiſted by opiates, are often adminiſtered in vain, and more eſpecially as the advantages we derive from theſe depend on the tendency they have to produce external gout. Whether æther be this ſpecific, I ſhall leave to future experiments to determine.

I have ſince that time frequently uſed it with the greateſt ſucceſs, and could adduce a great number of caſes to prove its efficacy in gouty affections of the ſtomach; but the above appears to me to be ſo much in point, as to make it unneceffary. I fhall, however, add one more, as it was attended with fome peculiar circumftances, and will corroborate what has been faid relative to the former cafe.

### CASE II.

J. W. Efq. of a fpare habit, but good conftitution, had, early in life, a commiffion in the army, and having indulged in the pleafures of the table, and offered frequent libations with others of his Bacchanalian friends, very much impaired his health, and had, on his face and other parts of the body, a great number of thofe eruptions generally termed fcorbutic. Before he was twenty-five years of age he had alfo had feveral attacks of an anomalous gout.

His appetite was, at length, totally gone, and himfelf fo weak and emaciated as to be fcarcely able to walk. For thefe complaints his phyfician fent him to try the efficacy of Bath waters. After previous evacuations, he drank them in fmall quantities for the fpace of three weeks, but with little advantage; for though he had pains in the feet at night, yet the weak efforts of nature were infufficient to accomplifh her purpofe, as he had a regular and conftant return of pain at the ftomach, attended tended with ficknefs, about five o'clock every morning.

Anti-arthritic medicines were prefcribed in various forms, without fuccefs.

Under thefe circumftances, he was defired to take forty drops of æther in fome peppermint water about an hour before the ufual return of pain, and repeat it at noon and at bed-time. The firft dofe gave fenfible relief; in five or fix days he was perfectly free from his ftomach complaint, and his cure was perfected by the Bath waters; though he drank them at intervals full three months.

If æther is capable of producing fuch powerful effects, what may not be expected from a genuine preparation of Hoffman's anodyne mineral liquor? To fuch as fuppofe that this medicine is every day ufed in the fhops, and fold by every chymift, this queftion may appear impertinent; but to thofe who know that Hoffman has given only loofe hints refpecting the compofition and preparation of it, the fubject may appear of more importance.

I think I need not fcruple to affert, that the preparation generally, nay univerfally, fold for Hoffman's mineral anodyne, is no other than an impure unrectified æther, which, as every one knows who is at all converfant with the fubject, confifts of fpirit of wine, æther, fome portion of the crude fulphureous acid, and a very fmall quantity of oleum dulce vitrioli. Chymifts have been, perhaps, partly led into this miftake from a remark of M. Macquer, who fays, "'Tis fuppofed that this anodyne "liquor is nothing more than this oil (ol. "dulce) diffolved and combined with the two "liquors, which afcend the firft in the diftilla-"tion, and which immediately precede the "acid fulphureous phlegm."

From whence this ingenious author drew this conclufion I know not, as Hoffman expreffly requires the oleum dulce to be diffolved in a pure fpirit *; and though he does not fay what fpirit is fpecifically meant, yet, from the properties he afcribes to it, it can be no other than pure æther †.

Oleum dulce vitrioli being confeffedly the bafis of this medicine, and æther its neceffary folvent,

* Obfervat. Phyfico-chym. Lib. ii. p. 495.

† "Hic quippe fpiritus, quem liquoris anodyni mineralis nomine infignire foleo, totus fulphureus eft, fubito et flagrantiffime ardet atque abfumitur et ocyffime à flamma candelæ, etiam tribus adhuc digitis remotus, flammam concipit, atque in

ſolvent, it only remains to determine the proportions and manner of preparation. — From a review of what Hoffman has ſaid, and the dim light he choſe to throw on this ſubject, I think I may preſume to give the following proceſs for its preparation: —

Take of pure æther by meaſure twenty-nine ounces;

Of ſweet oil of vitriol, (ſo called) and freſh drawn, one ounce by weight;

Add the oil to the ætherial ſpirit, and put the mixture into a chryſtal glaſs, the neck of which has been properly ground and fitted to a recipient — draw off the ſpirit by the moſt gentle degree of heat.

Hoffman's ability has been univerſally admitted, and his integrity, I believe, never diſputed; how valuable then ſhould we eſteem a medicine to which he has given ſuch unlimited praiſe in almoſt every part of his in calido conclavi ſubito in auras avolat; attactu tamen inſtar glacie eſt frigidiſſimus, ac probe deſtillatus et rectificatus, omni aquæ inſtar olei ſupernatat." *Vide* Medicinæ rationalis, Tom. iii. ſect. ii. De Sedantibus. — This deſcription, and theſe properties, are applicable to no fluid but pure æther.

works*!

works *! It is his univerfal anodyne † which he confidered as being poffeffed of fuch manifold virtues, and addreffes the Supreme Being with fo great a degree of fervour on account of the difcovery, as nothing could authorife but the fulleft conviction of its utility in practice, and the great degree of benefit accruing to mankind from its ufe.

It perhaps may not be improper to recite the virtues Hoffman attributes to his mineral anodyne liquor in a few particular cafes. He informs his readers that the ufe of this remedy is of very great latitude, and its virtues manifold; that it wonderfully relieves pain, and, like a charm, difpels thofe excruciating torments attendant on the cholic, ftone in the urinary bladder, and gout; that it fuddenly

* "Cujus virtutes in medendo mihi funt notiffimæ et "quas ego non fatis deprædicare poffum." — Obfervat. Chym.

† Speaking of opiates, he fays, "Ex quo tamen, divina "benignitas mihi conceffit inventionem liquoris penetrantis "et fragrantis aromatici faporis et odoris, ex ipfius vitrioli "portione fulphurea, quæ etiam à veteribus chymicis pro "anodyna habita eft, fingulari quadam chymica encheirefi "præparandi, ab *omnibus aliis tuto abftinui*." — De Sedantibus.

and efficaciously takes off spasm in whatever part of the body it may arise; is of very great service in hypochondriac and hysterical affections; in all epileptic and convulsive commotions; in violent vomitings; in the hickup and diarrhoea. Its first and chief action, he observes, is in the stomach; and he therefore recommends it as very efficacious in all diseases whose primary seat is in that organ, as hypochondriac inflation, nausea, heartburn, &c.; and indeed it has an incomparable virtue of expelling flatus. — With all these good qualities, he adds, it has none of the bad effects of opiates; but, on the contrary, the refreshing sleep it procures gives fresh vigour to the constitution. In fevers, attended with delirium, watchfulness, and debility, he says, it may be given without danger — "Vires enim "non consternit sed custodit."

He supposes it to excel all other medicines in the symptomatic epilepsy, and that it is most excellently calculated for relieving other convulsive motions. In nephritic complaints, when taken in small and repeated doses, he has found that it quiets the spasms in the first passages, takes off nausea and vomiting, and facilitates the discharge of gravel and stone.

I myfelf am fully perfuaded that, if fair and candid trials were made with the anodyne liquor juft now defcribed, Hoffman's account of it would not appear to be at all exaggerated, as it will be found to poffefs, in a much greater degree, all the virtues attributed to vitriolic æther.

*Queen Square, Bath,*

Aug. 23, 1785.

